# Nomogram individually predicts the risk for distant metastasis and prognosis value in female differentiated thyroid cancer patients: A SEER-based study

**DOI:** 10.3389/fonc.2022.800639

**Published:** 2022-08-10

**Authors:** Wenlong Wang, Cong Shen, Zhi Yang

**Affiliations:** ^1^ General Surgery Department, Xiangya Hospital, Central South University, Changsha, China; ^2^ National Clinical Research Center for Geriatric Disorders, Xiangya Hospital, Central South University, Changsha, China; ^3^ Department of Colorectal & Anal Surgery, Hepatobiliary & Enteric Surgery Research Center, Xiangya Hospital, Central South University, Changsha, China

**Keywords:** distant metastasis (DM), differentiated thyroid cancer (DTC), nomogram, overall survival (OS), prognosis

## Abstract

**Objective:**

Distant metastasis (DM) is an important prognostic factor in differentiated thyroid cancer (DTC) and determines the course of treatment. This study aimed to establish a predictive nomogram model that could individually estimate the risk of DM and analyze the prognosis of female DTC patients (FDTCs).

**Materials and methods:**

A total of 26,998 FDTCs were retrospectively searched from the Surveillance, Epidemiology, and End Results (SEER) database from 2010 to 2018 and randomly divided into validation and training cohorts. Univariate and multivariate analyses were performed to screen for prognostic factors and construct a prediction nomogram. The performance of the nomogram was assessed by the area under the receiver operating characteristic curve (AUC), concordance index (C-index), and a calibration curve. The overall survival (OS) and cancer-specific survival (CSS) were evaluated by Kaplan–Meier (K-M) analysis.

**Results:**

A total of 263 (0.97%) FDTCs were reported to have DM. K-M analysis showed the association of multiple-organ metastases and brain involvement with lower survival rates (*P* < 0.001) in patients. Tumor size, age at diagnosis, thyroidectomy, N1 stage, T3–4 stage, and pathological type were independent predictive factors of DM in FDTCs (all *P* < 0.001). Similarly, age at diagnosis, Black, DM, T3–4 stage, thyroidectomy, and lung metastasis were determined as independent prognostic factors for FDTCs (all *P* < 0.001). Several predictive nomograms were established based on the above factors. The C-index, AUC, and calibration curves demonstrated a good performance of these nomogram models.

**Conclusion:**

Our study was successful in establishing and validating nomograms that could predict DM, as well as CSS and OS in individual patients with FDTC based on a large study cohort. These nomograms could enable surgeons to perform individualized survival evaluation and risk stratification for FDTCs.

## Introduction

Thyroid cancer is one of the most commonly occurring endocrine malignancies, and its incidence has been dramatically increasing worldwide (1–3). Among the histological subtypes of thyroid cancer, differentiated thyroid cancer (DTC) accounts for nearly 90% of all thyroid carcinomas (4). DTC has a favorable prognosis, with a 10-year overall survival rate exceeding 90% (5). Nevertheless, a small number of DTC patients present with distant metastasis (DM) at initial diagnosis, which is the leading cause of thyroid cancer-related mortality (6, 7). The risk factors for DM have been widely discussed in previous studies, but the results were inconsistent in patients with DTC. Liu et al. (8) reported that larger tumor size, lymph node metastasis, male, and histological subtype of follicular thyroid cancer (FTC) significantly increased the risk of DM in patients with DTC. Huy et al. (9) demonstrated that vascular invasion, multifocality, and extrathyroidal extension were independent predictors of DM. Unfortunately, the above studies were retrospective, small-sampled, single-center studies and exhibited a huge heterogeneity. To date, there is no effective method to quantitatively predict DM in DTC patients.

Sex disparity in disease aggressiveness, prognosis, and incidence has been observed in a variety of cancers (10, 11). In thyroid cancer, women are two to three times more likely to develop DTC and present at an earlier stage of disease, than patients with a non-aggressive type of cancer or those diagnosed at a younger age (12). In contrast, men tend to have a more aggressive disease at diagnosis and ultimately suffer higher mortality and lower disease-free survival (13). Therefore, the potential impact of sex disparity on DM should be taken into consideration to reduce the influence of selection bias.

The SEER (https://seer.cancer.gov/) database is a large public database that represents approximately 28% of the US population and provides clinical information about patient demography, tumor morphology, diagnosis, treatment, and prognosis (14). Given the important role of DM in predicting survival outcomes in DTC patients, identifying the patients who have the possibility to develop DM and offering these individuals more aggressive treatments are paramount to achieving the best clinical outcomes. Nomogram is a simple and reliable statistical prediction tool that has been widely used in the clinical setting and is helpful for clinicians to recognize the high-risk female DTC patients (FDTCs) in a visual fashion.

Based on the heterogeneity of thyroid cancer, and multiple available treatment options, it is important to establish predictive models for DM and provide an appropriate therapeutic strategy for FDTCs. Moreover, there is a lack of studies focusing on developing a convenient and accurate risk assessment tool to predict the cancer-specific survival (CSS) and overall survival (OS) in FDTCs. To our knowledge, this is the first study attempting to use a large database to create a prognostic nomogram for FDTCs, which may enable personalized medical decision-making and surveillance more accurately.

## Materials and methods

### Data source

**Figure 1:**
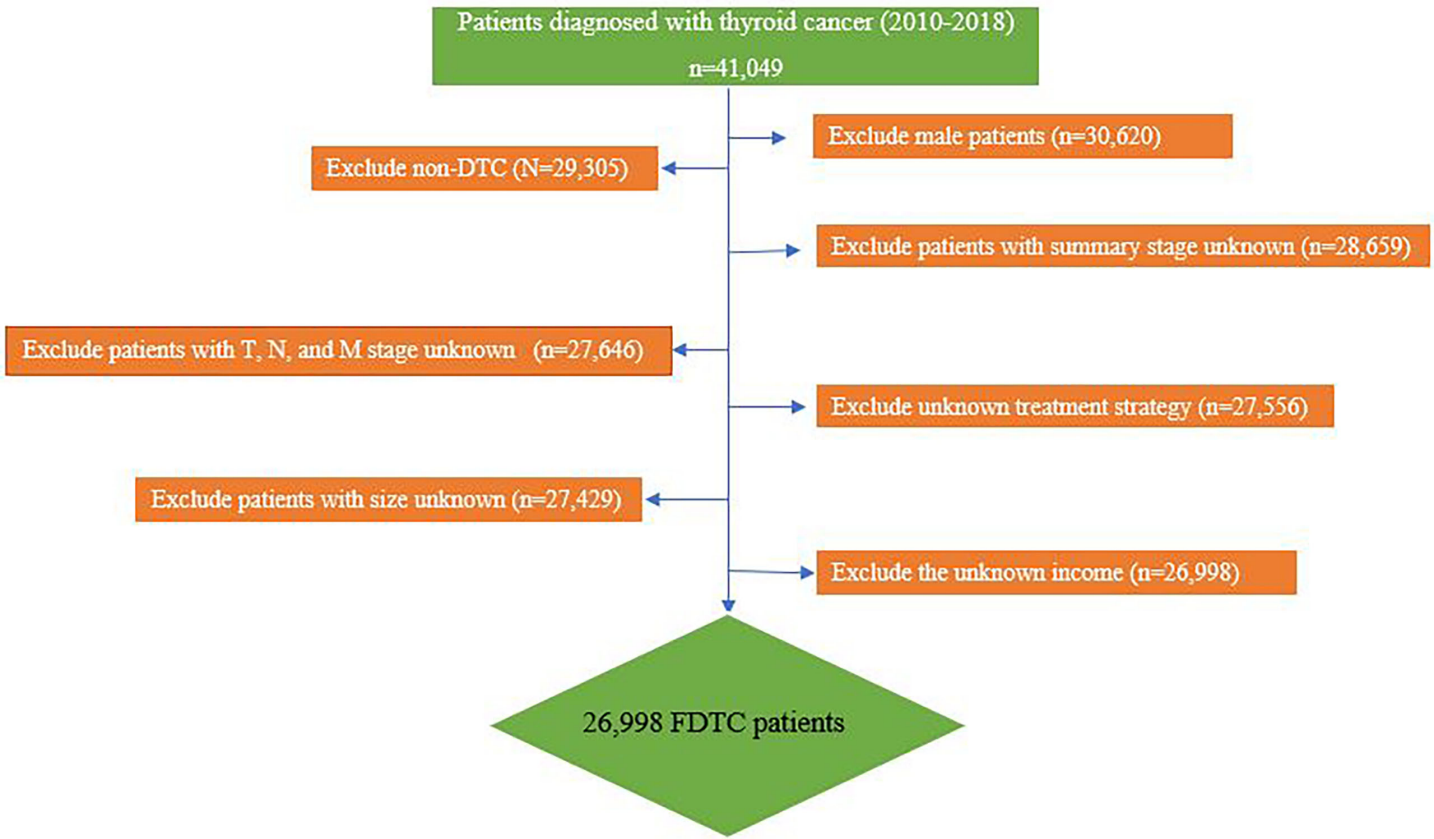
Research flowchart.

A retrospective cohort study was conducted by using selected data from the SEER database of the US National Cancer Institute. Our study was approved by the Ethics Committee of Xiangya Hospital of Central South University and conformed to the provisions of the Declaration of Helsinki. We extracted patients diagnosed with DTC (ICD-O-3 codes 8341, 8340,8050, 8260, 8050) from the SEER 21 region during the period 2010–2018. Information for metastatic sites of the lung (Combined Mets at DX-lung), bone (Combined Mets at DX-bone), liver (Combined Mets at DX-liver), and brain (Combined Mets at DX-brain) was collected since 2010. The inclusion criteria were as follows: (I) diagnosed with DTC at some time from 2010 to 2018; (II) female; (III) active follow-up during the study period; (IV) with a known cause of death. We excluded patients with insufficient or unknown clinicopathologic profile, other types, or undetermined histology of thyroid carcinomas. Finally, a total of 26,998 FDTCs were enrolled in the current study (**Figure 1**).

### Clinical characterization

The variables were extracted from the selected cohorts as follows: demographic variables included race (black, white, other) and age (<55 or ≥55 years); clinicopathological characteristics included T stage (Tx, T0, T1, T2, T3, and T4), N stage (Nx, N0, and N1), M stage (M0 or M1), TNM stage (I, II, III, and IV), summary stage (localized, regional, and distant), tumor size (≤10 or >10 mm), histology type (papillary, papillary with follicular variant, and follicular), median income (<$45,000, $45,000–$65,000, and ≥$65,000), survival months, and vital status. The endpoint of the current study was OS and CSS, which were defined as the duration from the initial diagnosis to all-cause death and the interval from the initial diagnosis to death from DTC, respectively (15). The seventh edition of the TNM classification system was used to stage FDTCs.

### Nomogram development

The nomogram was developed based on the results of multivariate analysis by using the “rms” package in R version 3.5.1 (http://www.r-project.org/). The performance of the nomogram was assessed by calibration and discrimination. A calibration plot (1,000 bootstrap resamples) was used to evaluate the discrimination of the model, the Harrell’s concordance index (C-index), ranging from 0.5 (indicates absence of discrimination) to 1 (indicates perfect discrimination) (16), which approximately equivalented to the area under the receiver operating characteristic curve (AUC). Furthermore, decision curve analysis (DCA) was employed to evaluate the clinical values and utility of the nomogram by R function “stdca” (17).

### Statistical analysis

Statistical analyses were performed in SPSS 22.0 (IBM Corp, USA). Categorical variables were presented as percentage (%), and continuous variables were expressed as the mean ± SD. Chi-square test and t test were used for categorical and continuous variables, respectively. Univariate and multivariate analyses were performed to identify the independent risk factors of DM or prognostic factors of CSS and OS. The Kaplan–Meier (K-M) method was employed to estimate the OS and CSS, and the significance of differences was assessed by log-rank tests. *P* value < 0.05 (two-sided) represented positive statistical significance.

## Results

### Clinicopathological features

**Figure 2:**
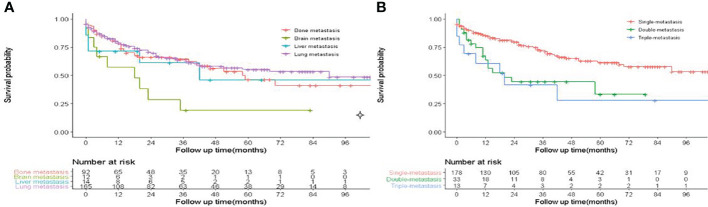
Effect of distant metastasis on overall survival in FDTCs. **(A)** The overall survival in different metastasis sites. **(B)** The overall survival of different metastasis patterns.

A total of 26,998 eligible FDTC patients from 2010 to 2018 were identified from the SEER database. The clinicopathological characteristics of the patients are displayed in **Table 1**. In the whole study cohort, 17,629 (65.3%) patients were younger than 55 years. The average age was 47.9 ± 15.13 years, and the majority of the patients were white (21,267, 78.8%) and had a larger tumor size (21,639, 80.2%). Of these, T1 stage (17,175, 63.6%), N0 stage (20,567, 76.2%), M0 stage (26,735, 99.0%), TNM I stage (21,072, 78.1%), and localized (19,039, 70.5%) were more common. Papillary (18,400, 68.2%) was the most common pathological subtype, followed by papillary with follicular variant (7,405, 27.4%) and FTC (1,193, 4.4%). A percentage of 98.4% patients had undergone thyroidectomy. The median follow‐up time was 50 months, during which a total of 918 (3.4%) patients had died and 0.65% (176/26,998) patients had died due to DTC. Besides, DM was found in 263 (0.97%) patients, of which 166 patients (0.61%) had lung metastases, 92 patients (0.34%) had bone metastases, 14 patients (0.05%) had liver metastases, and 12 patients (4.96%) had brain metastases. K-M analysis demonstrated that brain metastasis was associated with the worst survival than other metastasis sites (*P* < 0.001, **Figure 2A**). Compared with single metastasis, multiple metastases were associated with a poorer survival (*P* < 0.001). Interestingly, triple metastases did not reduce the survival time as compared to double metastases (**Figure 2B**). These results indicated that the pattern of organ-specific metastases had different prognostic values in FDTC.

**Table 1:** Characteristics of 26,998 patients with FDTC in SEER.

Variable	Patient demographics (%)
Age mean ± SD	47.9 ± 15.13
≥55 years	9,369 (34.7)
<55 years	17,629 (65.3)
Race	
White	21,267 (78.8)
Black	2,025 (7.5)
Other	3,706 (13.7)
Tumor size mean ± SD	7.58 ± 11.03
≤1.0 cm	5,359 (19.8)
>1.0 cm	21,639 (80.2)
T stage	
T0	25 (0.1)
Tx	125 (0.5)
T1	17,175 (63.6)
T2	4,481 (16.6)
T3	4,625 (17.1)
T4	567 (2.1)
N stage	
N0	20,567 (76.2)
Nx	445 (1.6)
N1	5,896 (22.2)
M stage	
M0	26,735 (99.0)
M1	263 (1.0)
TNM stage	
I	21,072 (78.1)
II	1,804 (6.7)
III	2,876 (10.7)
IV	1,246 (4.6)
Summary stage	
Localized	19,039 (70.5)
Regional	7,457 (27.6)
Distant	502 (1.9)
Thyroidectomy	26,575 (98.4)
Pathological type	
Papillary	18,400 (68.2)
Papillary with follicular variant	7,405 (27.4)
Follicular	1,193 (4.4)
Median income	
<$45,000	1,249 (4.6)
$45,000-$65,000	8,318 (30.8)
≥$65,000	17,431 (64.6)

FDTC, differentiated thyroid cancer; SEER, Surveillance, Epidemiology, and End Results.

### A novel nomogram predicting distant metastasis

Subsequently, 26,998 FDTC patients were randomly divided into validation and training cohorts (**Table S1**) to formulate and validate the nomogram. The univariate analysis indicated that the age at diagnosis, white, FTC, N1 stage, T3–4 stage, tumor size, thyroidectomy, and thyroid micro-carcinoma (TMC) were significantly associated with DM in the training cohorts (all *P*<0.05). Multivariate logistic regression analysis proved that the age at diagnosis, pathological type, N1 stage, T3–4 stage, thyroidectomy, and tumor size were independent risk factors for DM (all *P*<0.05, **Table 2**). In terms of the tumor size, an increasing DM risk was detected in patients with a larger tumor size (OR = 1.02, 95% CI = 1.01–1.04, *P* = 0.003). Also, an older age at diagnosis was associated with a higher risk of DM (OR = 1.03, 95% CI = 1.01–1.05, *P* = 0.001). Comparing papillary with follicular variant thyroid cancer, papillary thyroid cancer (PTC) (OR = 0.47, 95% CI = 0.3–0.73, *P<* 0.001) and FTC (OR = 4.73, 95% CI = 2.64–8.5, *P<* 0.001) showed an opposite effect. Thyroidectomy was associated with a significantly lower risk of DM (OR = 0.06, 95% CI = 0.03–0.09, *P<* 0.001). Furthermore, higher N stage (OR = 6.44, 95% CI = 4.21–9.84, *P<* 0.001) and T stage (OR = 2.66, 95% CI = 1.76–3.91, *P<* 0.001) were associated with a higher probability of DM.

**Table 2:** Univariate and multivariate logistics regression analyses of the risk factors of DM in the training cohorts.

Variable	Univariate analysis		Multivariate analysis	
	HR (95% CI)	*P*	OR (95% CI)	*P*
Age mean ± SD	1.06 (1.05-1.07)	<0.001	1.03 (1.01-1.05)	0.001
<55 years	Ref	<0.001	Ref	0.114
≥55 years	4.67 (3.24-6.72)		1.75 (0.87-3.51)	
Race				0.731
White	2.15 (1.13-4.09)	0.02	0.88 (0.42-1.84)	0.939
Black	0.88 (0.3-2.59)	0.82	0.96 (0.31-2.95)	
Other	Ref		Ref	
Pathological type				<0.001
Papillary with follicular variant	Ref	0.64	Ref	<0.001
Papillary	0.91 (0.61-1.36)	<0.001	0.47 (0.3-0.73)	
Follicular	5.04 (3.03-8.39)		4.73 (2.64-8.5)	
T stage				
T0-2	Ref	<0.001	Ref	<0.001
T3-4	5.46 (3.91-7.62)		2.62 (1.76-3.91)	
N stage				
N0/x	Ref	<0.001	Ref	<0.001
N1	3.82 (2.74-5.32)		6.44 (4.21-9.84)	
Thyroidectomy	0.05 (0.03-0.08)	<0.001	0.06 (0.03-0.09)	<0.001
Tumor size	1.04 (1.03-1.04)	<0.001	1.02 (1.01-1.04)	0.003
TMC	2.22 (1.57-3.14)	<0.001	0.69 (0.37-1.3)	0.254
Median income				
<$45,000,	Ref	0.89	Ref	–
$45,000-$65,000	1.07 (0.42-2.74)	0.38	–	–
≥$65,000	1.5 (0.61-3.69)		–	

CI, confidence intervals; TMC, thyroid microcarcinoma; DM, distant metastasis.

**Figure 3:**
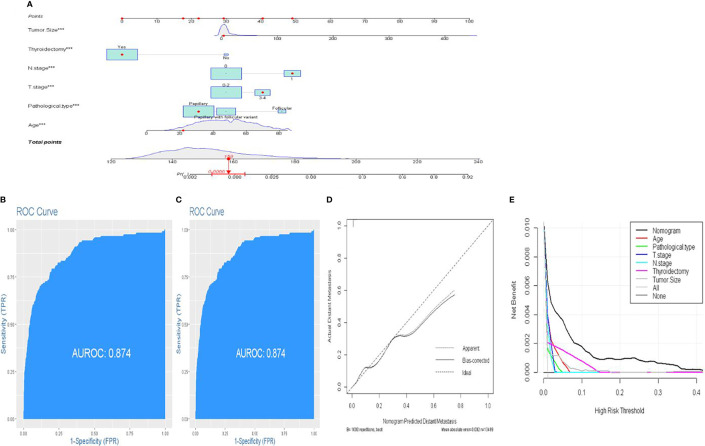
A novel nomogram predicting distant metastasis. **(A)** Nomogram predicting the probability of DM. Instructions: Locate the patient characteristics of each variable on its respective axis. Draw a line to the “dot” scale to get a score for each variable. Find the total score on the total score scale based on the sum of the points of all variables. Draw a line to the end scale. **(B, C)** The ROC curve of nomograms in training and validation cohorts. **(D)** Calibration curves of the nomogram for the probability of DM. **(E)** Decision curve analysis for nomogram.

In order to assess the risk variables of DM in FDTCs more intuitively, a nomogram model was constructed (**Figure 3A**). The C-index of nomogram for predicting DM was 0.887 in the training cohort and 0.878 in the validation cohort, respectively. The areas under the ROC curves (AUC) in the training and validation cohorts were 0.887 and 0.874, respectively (**Figures 3B** **, C**). The AUC combined with the C-index reflected a good discrimination ability of the nomogram. In addition, the calibration plots showed perfect consistency in both the training and validation cohorts (**Figure 3D**). Finally, DCA indicated that this nomogram had an excellent performance in clinical practice (**Figure 3E**).

### An individualized nomogram predicting bone metastasis and lung metastasis

**Figure 4:**
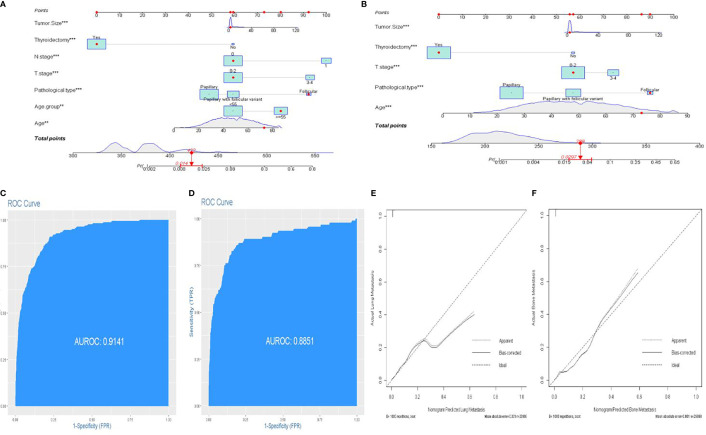
An individualized nomogram predicting bone metastasis and lung metastasis. **(A)** Nomogram predicting the probability of lung metastasis. **(B)** Nomogram predicting the probability of bone metastasis. **(C)** The ROC curve of the nomogram with lung metastasis. **(D)** The ROC curve of the nomogram with bone metastasis. **(E, F)** Calibration curves of the nomogram for the probability of lung metastasis and bone metastasis.

Lung and bone are the most common organs for metastasis in patients with DTC, and metastasis in these organs is known to correlate with a poor prognosis. Next, we developed two predictive nomograms to evaluate the possibility of bone and lung metastasis, respectively. Independent risk factors for bone and lung metastasis were identified by univariate and multivariate analyses. The age at diagnosis, FTC, N1 stage, T3–4 stage, tumor size, and thyroidectomy were identified as significant independent predictors of lung metastasis (all *P*<0.05, **Table 3**). Similarly, age at diagnosis, FTC, T3–4 stage, tumor size, and thyroidectomy were also identified as independent predictors of bone metastasis (all *P*<0.05, **Table 4**). These significant independent factors were incorporated to build a nomogram (**Figures 4A** **, B**). The C-index value of the nomogram for predicting lung and bone metastasis was 0.914 and 0.884, respectively. Meanwhile, the AUC of the nomogram for predicting lung and bone metastasis was 0.914 and 0.885, respectively (**Figures 4C** **, D**), which also *demonstrate*d the accuracy and reliability of the prediction model. The calibration curves showed excellent agreement between the predicted and actual observations (**Figures 4E** **, F**).

**Table 3:** Univariate and multivariate logistics regression analyses of the risk factors of lung metastasis in FDTC patients.

Variable	Univariate analysis		Multivariate analysis	
	HR (95% CI)	*P*	OR (95% CI)	*P*
Age mean ± SD	1.07 (1.05-1.08)	< 0.001	1.02 (1.00-1.04)	0.018
<55 years	Ref	< 0.001	Ref	0.005
≥55 years	5.95 (3.55-9.77)		2.5 (1.31-4.74)	
Race				0.687
White	3.37 (1.66-6.87)	< 0.001	1.18 (0.54-2.58)	0.429
Black	0.69 (0.18-2.59)	0.578	0.58 (0.15-2.24)	
Other	Ref		Ref	
Pathological type				0.033
Papillary with follicular variant	Ref	0.414	Ref	< 0.001
Papillary	1.17 (0.8-1.72)	< 0.001	0.64 (0.42-0.96)	
Follicular	5.06 (3.07-8.35)		4.53 (2.59-7.93)	
T stage				
T0-2	Ref	< 0.001	Ref	< 0.001
T3-4	9.05 (6.52-12.56)		4.57 (3.17-6.61)	
N stage				
N0/x	Ref	< 0.001	Ref	< 0.001
N1	5.07 (3.72-6.93)		6.35 (4.33-9.31)	
Thyroidectomy	0.05 (0.03-0.07)	< 0.001	0.07 (0.04-0.11)	< 0.001
Tumor size	1.03 (1.02-1.04)	< 0.001	1.01 (1.00-1.02)	0.004
TMC	2.03 (1.47-2.81)	< 0.001	0.88 (0.55-1.41)	0.591
Median income				
<$45,000,	Ref	0.571	Ref	–
$45,000-$65,000	1.28 (0.55-2.98)	0.543	–	–
≥$65,000	1.29 (0.57-2.94)		–	

CI, confidence intervals; TMC, thyroid microcarcinoma; FDTC, female differentiated thyroid cancer.

**Table 4:** Univariate and multivariate logistics regression analyses of the risk factors of bone metastasis in FDTC patients.

Variable	Univariate analysis		Multivariate analysis	
	HR (95% CI)	*P*	OR (95% CI)	*P*
Age mean ± SD	1.08 (1.07-1.1)	< 0.001	1.04 (1.01-1.06)	0.005
<55 years	Ref	< 0.001	Ref	0.149
≥55 years	8.36 (4.94-14.16)		1.87 (0.8-4.38)	
Race				0.141
White	15.57 (2.17-111.67)	0.006	4.58 (0.6-34.8)	0.254
Black	3.66 (0.33-40.38)	0.289	4.12 (0.36-46.92)	
Other	Ref		Ref	
Pathological type				< 0.001
Papillary with follicular variant	Ref	< 0.001	Ref	< 0.001
Papillary	0.42 (0.25-0.7)	< 0.001	0.31 (0.17-0.54)	
Follicular	7.96 (4.83-13.14)		4.74 (2.76-8.15)	
T stage				
T0-2	Ref	< 0.001	Ref	< 0.001
T3-4	3.71 (2.46-5.59)		2.24 (1.4-3.61)	
N stage				
N0/x	Ref	0.687	–	–
N1	1.1 (0.68-1.78)		–	
Thyroidectomy	0.05 (0.03-0.07)	< 0.001	0.06 (0.04-0.12)	< 0.001
Tumor size	1.03 (1.02-1.04)	< 0.001	1.02 (1.00-1.03)	0.015
TMC	1.86 (1.2-2.9)	0.006	0.81 (0.39-1.69)	0.581
Median income				
<$45,000,	Ref	0.56	Ref	- -
$45,000-$65,000	1.65 (0.39-7.04)	0.214	–	
≥$65,000	2.44 (0.6-9.97)		–	

CI, confidence intervals; TMC, thyroid microcarcinoma; FDTC, female differentiated thyroid cancer.

### A prognostic nomogram predicting OS and CSS

**Figure 5:**
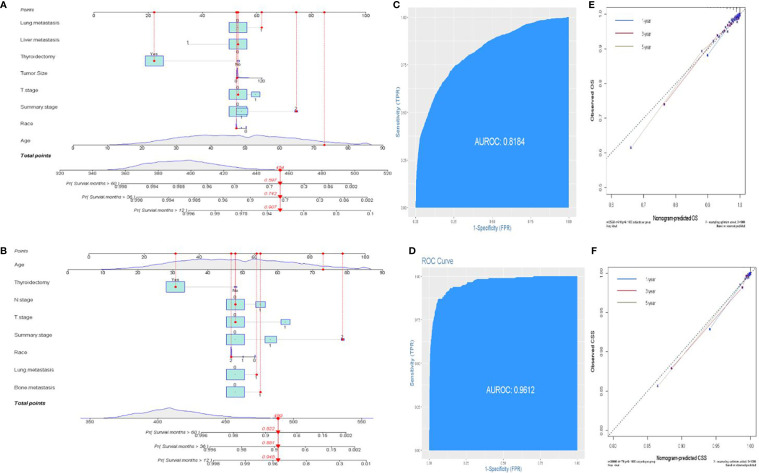
Establishment of prognostic nomograms. **(A, B)** Nomogram predicting the probability of OS and CSS. **(C, D)** The ROC curve of the nomogram with OS and CSS. **(E, F)** Calibration curves of the nomogram for the probability of OS and CSS.

The prognostic significance of gender is still controversial, and there is no clinical evidence relating gender with prognosis in FDTCs. To identify the independent prognostic factors associated with the survival of FDTCs, univariate and multivariate Cox analyses were performed. Statistical analyses demonstrated that the age at diagnosis (*P*<0.001), Black (*P* = 0.008), distant metastasis (*P*<0.001), T3–4 stage (*P*<0.001), thyroidectomy (*P*<0.001), tumor size (*P* = 0.044), lung metastasis (*P* = 0.005), and liver metastasis (*P* = 0.009) were independent risk factors for OS (**Table 5**). Moreover, in the CSS analysis, the age at diagnosis (*P*<0.001), Black (*P* = 0.043), regional (*P* = 0.004), distant metastasis (*P*<0.001), T3–4 stage (*P*<0.001), N1 stage (*P*<0.001), thyroidectomy (*P*<0.001), lung metastasis (*P* = 0.003), and bone metastasis (*P* = 0.009) were significantly associated with CSS (**Table 6**). The OS and CSS nomograms were constructed based on the independent prognostic factors (**Figures 5A** **, B**) and were validated internally. The C-index for the OS nomogram was 0.826, while it was 0.827 for the CSS nomogram. The ROC is plotted in **Figure 2**, and the AUCs for OS and CSS were 0.818 and 0.961, indicating acceptable discriminations (**Figures 5C** **, D**). In addition, the calibration curves indicated an excellent agreement between the actual survival and the predicted results (**Figures 5E** **, F**).

**Table 5:** Univariate and multivariate cox regression analyses of the prognostic factors of OS in FDTC patients.

Variable	Univariate analysis		Multivariate analysis	
	HR (95% CI)	*P*	OR (95% CI)	*P*
Age mean ± SD	1.09 (1.08-1.11)	< 0.001	1.08 (1.07-1.09)	< 0.001
<55 years	Ref	< 0.001	Ref	0.542
≥55 years	6.9 (5.9-8.06)		0.92 (0.71-1.2)	
Race				0.625
White	3.28 (2.41-4.47)	< 0.001	0.92 (0.65-1.29)	0.008
Black	1.1 (0.67-1.81)	0.714	2.01 (1.2-3.37)	
Other	Ref		Ref	
Pathological type				
Papillary with follicular variant	Ref	0.419	–	–
Papillary	1.06 (0.92-1.23)	0.006	1.01 (0.87-1.17)	0.916
Follicular	1.5 (1.12-2.01)		0.75 (0.54-1.04)	0.084
Summary stage				0.589
Localized	Ref	0.11	Ref	< 0.001
Regional	1.13 (0.97-1.32)	< 0.001	1.06 (0.87-1.28)	
Distant	12.51 (10.42-15.02)		3.24 (2.44-4.32)	
T stage				
T0-2	Ref	< 0.001	Ref	<0.001
T3-4	2.29 (2–2.63)		1.47 (1.23-1.77)	
N stage				
N0/x	Ref	0.063	–	–
N1	1.16 (0.99-1.35)		–	
Thyroidectomy	0.06 (0.05-0.07)	< 0.001	0.18 (0.14-0.22)	< 0.001
Tumor size	1.01 (1.01-1.02)	< 0.001	1.0 (1.01-1.01)	0.044
TMC	1.31 (1.02-1.68)	0.034	0.93 (0.68-1.26)	0.62
Median income				
<$45,000,	Ref	0.157	Ref	- -
$45,000-$65,000	0.82 (0.62-1.08)	0.059	–	
≥$65,000	0.73 (0.56-0.95)		–	
Lung metastasis	17.09 (13.15-22.21)	< 0.001	1.65 (1.16-2.33)	0.005
Bone metastasis	20.53 (14.83-28.43)	< 0.001	1.29 (1.16-2.33)	0.262
Liver metastasis	22.79 (10.21-50.88)	< 0.001	0.3 (0.12-0.74)	0.009
Brain metastasis	55.88 (28.94-107.9)	< 0.001	1.53 (0.72-3.23)	0.264

CI, confidence intervals; TMC, thyroid microcarcinoma; FDTC, female differentiated thyroid cancer; OS, overall survival.

**Table 6:** Univariate and multivariate cox regression analyses of the prognostic factors of CSS in FDTC patients.

Variable	Univariate analysis		Multivariate analysis	
	HR (95% CI)	*P*	OR (95% CI)	*P*
Age mean ± SD	1.14 (1.11-1.16)	< 0.001	1.09 (1.07-1.11)	< 0.001
<55 years	Ref	< 0.001	Ref	0.686
≥55 years	16.99 (10.43-27.66)		1.17 (0.54-2.54)	
Race		< 0.001		0.53
White	6.83 (2.53-18.42)	0.108	0.7 (0.23-2.15)	0.043
Black	2.82 (0.8-10.01)		3.88 (1.04-14.43)	
Other	Ref		Ref	
Pathological type				
Papillary with follicular variant	Ref	0.007	Ref	–
Papillary	1.72 (1.16-2.55)	< 0.001	1.01 (0.66-1.53)	0.979
Follicular	4.98 (2.9-8.54)		1.18 (0.61-2.3)	0.621
Summary stage		< 0.001		0.004
Localized	Ref	< 0.001	Ref	< 0.001
Regional	6.54 (4.03-10.61)		2.4 (1.32-4.36)	
Distant	184.63 (117.1-191.11)		13.05 (6.87-24.8)	
T stage		< 0.001		< 0.001
T0-2	Ref		Ref	
T3-4	12.55 (8.92-17.65)		3.46 (2.26-5.3)	
N stage		< 0.001		< 0.001
N0/x	Ref		Ref	
N1	4.56 (3.38-6.13)		1.93 (1.34-2.77)	
Thyroidectomy	0.03 (0.02-0.04)	< 0.001	0.2 (0.13-0.31)	< 0.001
Tumor size	1.01 (1.01-1.02)	< 0.001	1.01 (1.01-1.02)	0.057
TMC	2.18 (1.25-3.78)	0.006	2.18 (1.25-3.78)	0.868
Median income				
<$45,000,	Ref	0.996	Ref	–
$45,000-$65,000	1 (0.35-2.88)	0.825	–	–
≥$65,000	1.12 (0.41-3.09)		–	
Lung metastasis	80.28 (57.41-112.3)	< 0.001	1.97 (1.26-3.09)	0.003
Bone metastasis	79.79 (53.45-119.1)	< 0.001	2.22 (1.22-4.04)	0.009
Liver metastasis	86.99 (35.7-212.0)	< 0.001	0.37 (0.13-1.06)	0.065
Brain metastasis	178.2 (83.33-381.2)	< 0.001	0.59 (0.22-1.6)	0.301

CI, confidence intervals; TMC, thyroid microcarcinoma; FDTC, female differentiated thyroid cancer; CSS, cancer-specific survival.

## Discussion

Overall, DM is uncommon in DTC, and the lung is the most common site for metastasis, followed by the bone, liver, brain, and kidney (18, 19). Thyroid cancer patients who develop DM are reported to have a poor prognosis and low overall survival (20). During the past decades, many studies have focused on identifying the heterogeneous and homogeneous prognosis-related factors of DM, including male gender, tumor size, histology subtype, extrathyroidal extension, age at diagnosis, and lymph node metastasis status (6, 8, 21–24). However, the potential impact of gender disparity on thyroid cancer prognosis was ignored. To the best of our knowledge, our study is the first large-scale analysis that has established an individual nomogram model to assess the prognosis of FDTCs and quantify the risk of DM, specifically regarding lung metastasis and bone metastasis. Our nomogram models would provide a personalized and accurate tool for the clinicians to determine more effective and reasonable treatment strategies for FDTCs.

In the present study, DM was found in 263 (0.97%) patients and the lung (166, 0.61%) was the most frequent organ of metastasis, which was consistent with the previous studies (18, 25, 26). The pathogenesis of metastasis still remains unclear, and DM is known to correlate with a higher mortality rate. In this study, we sought to investigate the effect of different DM sites on survival in patients with DTC to better understand their association with survival outcomes. Our results showed that brain metastasis was associated with a poorer survival rate than the other metastasis sites. Moreover, multiple-organ metastases had a poorer survival compared to single-organ metastases (p < 0.001). Interestingly, triple-organ metastases did not reduce the OS rate and CSS rate, as compared to double-organ metastases. This can be attributed to the rare incidence of synchronous DM, and majority of DM developed during the clinical follow-up. This is also partially because early identification of DM at the initial diagnosis is difficult, which may lead to an incomplete registration in the SEER database.

In the recent decades, different studies have attempted to identify the risk factors of DM in patients with DTC. A meta-analysis demonstrated male, older age, extrathyroidal extension, vascular invasion, and lymph node metastasis to be significant risk factors for DM (9). The study by Kwon et al. (27). reported radiomics analysis based on grayscale ultrasound to predict DM. Nevertheless, there have been no published studies for predicting DM in female patients with DTC. To our knowledge, this is the first study attempting to identify the risk factors and create a nomogram model for recognizing early DM in FDTCs. Our study showed that the age at diagnosis, FTC, N1 stage, T3–4 stage, tumor size, and thyroidectomy were significantly associated with DM. Therefore, this discrepancy should be taken into consideration when formulating treatment strategies. Lungs and bone are the most common sites for metastasis, which have been reported to be associated with a poor prognosis. Therefore, we investigated the risk factors for bone metastasis and lung metastasis in order to facilitate the early detection of metastatic lesions. We found that the age at diagnosis, FTC, T3–4 stage, and a larger tumor size were correlated with the development of bone or lung metastasis, which was consistent with previous research (28, 29). FDTCs with these risk variables should be monitored closely during follow-up. More importantly, to improve risk stratification and enable personalized medical decision-making, we developed and validated a predictive nomogram to identify high-risk patients with ease. Meanwhile, this predictive model showed excellent performance in assessing the risk for DM in FDTCs.

Although FDTC has a favorable prognosis, about 30% of the patients experience relapse and metastasis, which indicates the need for an accurate assessment of their prognosis (30). AGES, MACIS, AMES scoring system, and the TNM staging system have been frequently employed for the prognostic stratification of cancer patients (31). However, no scoring system could comprehensively and accurately assess the prognosis of patients of DTC, especially for FDTCs. In the present study, we identified that the age at diagnosis, Black, distant metastasis, T3–4 stage, thyroidectomy, and lung metastasis were independent prognostic factors for OS and CSS in FDTCs. Older age has been widely reported to be associated with a poor prognosis (32, 33). Advanced age was associated with an increased mortality rate. T3–4 stage was an independent prognostic factor whereas the tumor size was not; this might be because the T stage represents the extent of the primary tumor, including the extrathyroidal extension and tumor size. Wen et al. (34)demonstrated that DTC patients that underwent thyroidectomy had improved survival rates, similar to the results from our study. Moreover, DM had a significant adverse impact on the mortality rate, wherein the presence of bone and lung metastasis significantly reduced the FDTC-specific survival rate (*P* < 0.001). Subsequently, we constructed a nomogram model in order to predict the survival rate more accurately. The current nomogram may enable clinicians to identify high-risk FDTCs with a poor prognosis, so that such patients could be provided follow-up surveillance and better therapeutic strategies.

However, the current study had a few limitations. In the first place, the SEER database had some inherent limitations, including the lack of availability of information regarding some of the critical prognostic factors, such as the extent of surgery, margin status, radioiodine dosage, and BRAF V600E. Prognostic factor analysis based on the SEER database was incomplete. Therefore, the nomogram provided a relative clinical guidance value for clinicians and needed to be further improved after adding these relevant data. Besides, we adopted the seventh edition of the TNM classification system to stage FDTCs. The latest edition raised the cutoff age from 45 to 55 years and removed the microscopic extra-thyroidal extension from the T3 disease; these differences affect the accuracy of the nomogram. Third, a total of 26,998 FDTCs were enrolled from 2010 to 2018, leading to some data missing, because the SEER database recorded the metastatic sites from 2010. Furthermore, the novel predictive model was only validated internally; external validation was still necessary. Considering the above limitations, further randomized controlled trials should be recommended to improve the current prognostic nomogram.

In summary, the pattern of distant metastatic organ involvement was associated with variability in CSS and OS in FTDCs. We successfully established and validated nomograms to predict DM, including lung metastasis and bone metastasis, as well as CSS and OS in individual FTDCs, based on a large study cohort. Although some limitations exist in this predictive model, our nomograms provide a personalized, convenient, and visual clinical tool for the assessment of prognosis and risk for DM, which may enable surgeons to conduct individualized survival evaluation and identify the risk for DM in FDTCs.

## Data availability statement

The original contributions presented in the study are included in the article/**Supplementary Material**. Further inquiries can be directed to the corresponding author.

## Ethics statement

The studies involving human participants were reviewed and approved by ur study was approved by the Ethics Committee of Xiangya Hospital of Central South University and conformed to the provisions of the Declaration of Helsinki. The patients/participants provided their written informed consent to participate in this study.

## Author contributions

WW and ZY designed the study. WW and CS conducted the statistical analysis. WW and CS collected the clinical data. ZY wrote the whole paper. ZY and CS supervised and edited the paper. All authors contributed to the article and approved the submitted version.

## Funding

This work was supported by the Innovative Foundation for graduate students of Hunan Province (grant No. 2020zzts259).

## Conflict of interest

The authors declare that the research was conducted in the absence of any commercial or financial relationships that could be construed as a potential conflict of interest.

## Publisher’s note

All claims expressed in this article are solely those of the authors and do not necessarily represent those of their affiliated organizations, or those of the publisher, the editors and the reviewers. Any product that may be evaluated in this article, or claim that may be made by its manufacturer, is not guaranteed or endorsed by the publisher.

## Abbreviations

DM, distant metastasis; FDTC, female differentiated thyroid cancer; AUC, the area under the receiver operating characteristic curve; C-index, concordance index; OS, overall survival; CSS, cancer-specific survival; K-M, Kaplan–Meier; FTC, follicular thyroid cancer; SEER, Surveillance, Epidemiology, and End Results; DCA, decision curve analysis; TMC, thyroid microcarcinoma.
