# Supplementary material for: Nomogram individually predicts the risk for distant metastasis and prognosis value in female differentiated thyroid cancer patients: A SEER-based study
Source: Front Oncol. 2022 Aug 10;12:800639. doi: 10.3389/fonc.2022.800639 (PMC9399418; doi:10.3389/fonc.2022.800639)
Supplement: Supplementary file 1 [file Table_1.docx]

Table S1: Clinical and pathological features of FDTC patients.

| Variable | **Train cohort (N=13499)** | **Valid cohort (N=13499)** |
| --- | --- | --- |
| **Age (years)** |  |  |
| Mean (SD) | 47.9 (15.5) | 47.9 (15.4) |
| Median [Min, Max] | 47.0 [5.00, 85.0] | 47.0 [3.00, 85.0] |
| **Age. group (years)** |  |  |
| <55 | 8784 (65.1%) | 8845 (65.5%) |
| >=55 | 4715 (34.9%) | 4654 (34.5%) |
| **Race** |  |  |
| Other | 1795 (13.3%) | 1911 (14.2%) |
| Black | 1015 (7.5%) | 1010 (7.5%) |
| White | 10689 (79.2%) | 10578 (78.4%) |
| **Pathological type** |  |  |
| Papillary with follicular variant | 3679 (27.3%) | 3726 (27.6%) |
| Papillary | 9235 (68.4%) | 9165 (67.9%) |
| Follicular | 585 (4.3%) | 608 (4.5%) |
| **Summary stage** |  |  |
| Localized | 9473 (70.2%) | 9566 (70.9%) |
| Regional | 3756 (27.8%) | 3701 (27.4%) |
| Distant | 270 (2.0%) | 232 (1.7%) |
| **T stage** |  |  |
| 0-2 | 10865 (80.5%) | 10941 (81.1%) |
| 3-4 | 2634 (19.5%) | 2558 (18.9%) |
| **N stage** |  |  |
| 0 | 10461 (77.5%) | 10551 (78.2%) |
| 1 | 3038 (22.5%) | 2948 (21.8%) |
| **M stage** |  |  |
| 0 | 13357 (98.9%) | 13378 (99.1%) |
| 1 | 142 (1.1%) | 121 (0.9%) |
| **Thyroidectomy** |  |  |
| No | 205 (1.5%) | 218 (1.6%) |
| Yes | 13294 (98.5%) | 13281 (98.4%) |
| **Tumor Size (mm)** |  |  |
| Mean (SD) | 7.79 (11.7) | 7.38 (10.3) |
| Median [Min, Max] | 3.00 [0, 444] | 3.00 [0, 108] |
| **TMC** |  |  |
| No | 10760 (79.7%) | 10879 (80.6%) |
| Yes | 2739 (20.3%) | 2620 (19.4%) |
| Median Income **($)** |  |  |
| <45000 | 636 (4.7%) | 613 (4.5%) |
| 45000~65000 | 4162 (30.8%) | 4156 (30.8%) |
| >=65000 | 8701 (64.5%) | 8730 (64.7%) |

Abbreviations: CI: confidence intervals, HR: Hazard Ratio, TMC：thyroid microcarcinoma.
